# CAR Selectively Enhances the Pulmonary Vasodilatory Effect of Fasudil in a Microsphere Model of Pulmonary Hypertension

**DOI:** 10.2174/18743064-v17-e230404-2022-19

**Published:** 2023-04-27

**Authors:** Abraham Rothman, Humberto Restrepo, William N. Evans, Valeri Sarukhanov, David Mann

**Affiliations:** 1 Children’s Heart Center, Nevada 3131 La Canada, Suite 230, Las Vegas, NV 89169, USA; 2 Department of Pediatrics, University of Nevada Las Vegas, School of Medicine, 2040 W Charleston Blvd Ste. 402, Las Vegas, NV 89109, USA; 3 Vascular BioSciences, 72 Santa Felicia Dr, Goleta, CA 93117, USA

**Keywords:** Animal research, CAR, Homing peptide, Fasudil, Pulmonary hypertension, Swine

## Abstract

**Background:**

Despite the approval of several medications for pulmonary hypertension, morbidity and mortality are unacceptably high. Systemic hypotension may limit the use of pulmonary hypertension medications.

**Objectives:**

This study aimed to assess whether the homing peptide CAR (CARSKNKDC) improves the vasodilatory selectivity of fasudil in the pulmonary circulation or systemic circulation in a porcine pulmonary hypertension model.

**Materials and Methods:**

Pulmonary hypertension (to approximately 2/3-3/4 systemic pressure levels) was induced by chronic and acute administration of microspheres in 3 micro Yucatan pigs (mean weight 19.9 kg, mean age 4.3 months). Fasudil (0.3 mg/kg) was administered without and with CAR (1.5 mg/kg), and the effect on aortic (Ao) and right ventricular (RV) pressure was recorded with indwelling catheters.

**Results:**

Immediately after fasudil administration, there was a decrease in Ao pressure followed by prompt recovery to baseline. The RV pressure decrease was progressive and sustained. Fasudil alone resulted in a 12% decrease in RV pressure, whereas co-administration of CAR with fasudil resulted in a 22% decrease in RV pressure (p < 0.0001). Fasudil alone caused an average decrease of 34% in the RV/Ao pressure ratio, and fasudil + CAR caused an average decrease of 40% in the RV/Ao pressure ratio (p < 0.0001).

**Conclusion:**

The homing peptide CAR selectively enhanced the acute vasodilatory effects of fasudil on the pulmonary vascular bed in a porcine experimental model of pulmonary hypertension.

## 1 INTRODUCTION

Pulmonary hypertension frequently leads to right ventricular failure and death [1, 2]. Over the last three decades, several medications have received approval for treating pulmonary hypertension; nevertheless, systemic hypotension and additional adverse effects may limit their use. Pharmacologically enhancing the selectivity of vasoactive agents on the pulmonary vascular bed might be additionally beneficial for enhancing the vasodilatory effect and reducing side effects. CAR (CARSKNKDC) is a cyclic peptide reported to improve the selective pulmonary vasodilatory action of sildenafil, imatinib, and fasudil in rats with Sugen 5416/hypoxia/normoxia-induced pulmonary hypertension [3]. In this study, we aimed to extend these findings to different animal species and a model of pulmonary hypertension, which closely mimics pulmonary thromboembolic disease in humans. We evaluated whether CAR enhanced the acute pulmonary vasodilatory effects of fasudil, a Rho kinase inhibitor, in a large animal (porcine) microsphere model of pulmonary hypertension.

## 2 MATERIALS AND METHODS

The study conformed to the Guide for the Care and Use of Laboratory Animals [4]. The University of Nevada Las Vegas Institutional Animal Subjects Committee approved this study, and three Micro Yucatan female swine (Sinclair Research Center, Windham ME) were used to test fasudil (*Fasudil HCL)* in a chronic and acute model of pulmonary hypertension. The animals had a body weight of 10.5, 24.0, and 25.3 kg and an age of 2, 5, and 6 months, respectively, at the time of baseline cardiac catheterization.

### 2.1 Sedation

Pre-anesthesia consisted of a mixture of ketamine hydrochloride (Ben Venue Laboratories, Inc., Bedford, OH) 22 mg/kg, acepromazine (Vedco, Inc. St Joseph, MO) 0.2 mg/kg, and atropine (Baxter Healthcare Corp, Deerfield, IL) 0.05 mg/kg, intramuscularly. Induction and general anesthesia were attained with inhaled isoflurane (Baxter Healthcare Co., Deer Field, IL) 0.5-2.0%. Fentanyl 0.03-0.05 mg/kg was given for the maintenance of anesthesia. The animals were ventilated at a rate of 12-18 breaths/minute. One gram of cefazolin (West-Ward Pharmaceutical Corp., Eatontown, NJ) was administered intramuscularly before the procedure and 12 hours later. Postoperatively, animals received furosemide orally (Vintage Pharmaceuticals, LLC, Huntsville, AL) of 2-4 mg/kg twice daily for 2-3 days, cephalexin (Teva Pharmaceuticals, North Wales, PA) capsules of 500 mg twice a day for 2 days, and buprenorphine (Reckitt Benckiser Pharmaceutical, Parsippany, NJ) of 0.01-0.02 mg/kg twice daily for pain for 3 days and then as needed if the animals showed signs of discomfort. No acute or chronic anticoagulant or antiplatelet drugs were given.

### 2.2 Procedure

**Fig. (1):**
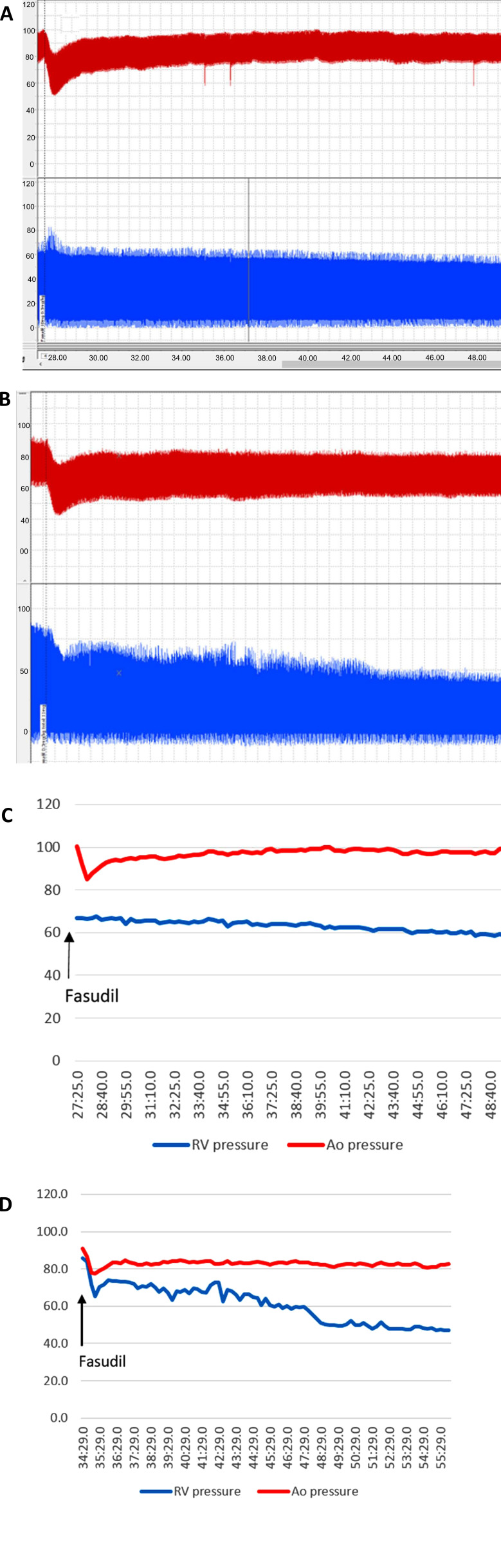
Representative PowerLab and derived curves. **Legend**: Ao (red) and RV (blue) pressure tracings were obtained simultaneously on PowerLab after the administration of fasudil (0.3 mg/kg) alone (**A**) and fasudil after CAR (1.5 mg/kg) (**B**). From the PowerLab tracings, curves were generated denoting the systolic Ao (red) and systolic RV (blue) pressures (**C** and **D**, respectively). Time is on the X-axis, in minutes and seconds. Pressure is on the Y-axis, in mmHg. Ao = Aortic, RV= Right Ventricular.

Access was attained *via* the carotid artery (5F sheath) and an external jugular vein (8F sheath), isolated by cutdown. A 7F wedge catheter was used to obtain pressures in the right atrium, right ventricle (RV), and pulmonary arteries. The pulmonary hypertensive model was developed by chronic delivery of ceramic microspheres in addition to acute administration of microspheres on the day of the vasodilator experiments (see below). Millar catheters (ADInstruments Inc., Colorado Springs, CO, USA) were inserted through the arterial sheath into the aortic (Ao) arch and the venous sheath into the RV. Pressures were recorded using a PowerLab C machine and software (AD Instruments Inc., Colorado Springs, CO, USA) (Fig. **1**). At the end of the procedure, the artery and vein were repaired using a microsurgical technique, with the aim of reusing these vessels in subsequent experiments.

### 2.3 Hypertensive Model Creation

To develop the hypertensive model, ceramic microspheres (3M, St Paul, MN), with a mean diameter of 0.6-0.9 mm, were delivered approximately every 2-3 weeks through an 8F sheath placed in the jugular vein. Microspheres (300 to 500 milligrams) were infused until the peak RV systolic pressure was 5 to 10 mmHg higher than the maximum level obtained during the previous infusion procedure. While the baseline RV pressure increased only modestly (see Results section) after multiple microsphere infusions, it was possible to acutely increase the RV systolic pressure to approximately 2/3-3/4 of the systemic level and perform acute vasodilator studies. The systemic and RV pressures were recorded at baseline (prior to fasudil or CAR infusion), for 5 minutes after the infusion of CAR, and for 20 minutes after the infusion of fasudil.

### 2.4 Fasudil and CAR

An initial dose-response study on fasudil was performed with three different doses, 0.1 mg/kg, 0.3 mg/kg, and 1.0 mg/kg, aiming for less than a 10% drop in Ao pressure. Each experiment was repeated three times. The homing cyclic peptide CARSKNKDC (CAR) (Vascular BioSciences, Goleta, CA) was administered at a dose of 1.5 mg/kg three times to assess the effects of CAR alone. Subsequently, in separate experimental days, fasudil was given alone, or fasudil was given 5 minutes after the administration of CAR. Ao and RV pressures were recorded continuously.

### 2.5 Statistical Analysis

The data were analyzed by comparing the decrease in RV pressure in relation to baseline and to the change in Ao pressure (RV/Ao ratio) for the days when fasudil was given alone vs. the days when fasudil was given after CAR. A t-test for paired mean samples and for the difference of the mean of the areas under the curves was performed. A p-value < .05 was considered significant.

### 3 RESULTS

#### 3.1 Model Creation

After a baseline catheterization, ceramic microsphere infusions were performed every 2-3 weeks. The intended RV pressure at each catheterization procedure was about 5-10 mmHg higher than the previous procedure. The baseline RV pressure at subsequent catheterizations, prior to new microsphere infusions, increased only modestly (consistently to about 30-35% of the systemic level), despite more than 6 months of repeated microsphere infusions. However, after several microsphere infusion procedures, with an acute infusion of microspheres at a single catheterization, an RV systolic pressure of about 2/3-3/4 systemic level could be achieved. It was at this level of RV pressure that the fasudil and CAR experiments were performed.

#### 3.2 Dose-Response

The 0.1 mg/kg dose of fasudil resulted in no significant decrease in Ao pressure and a 5% decrease in pulmonary artery pressure. The 0.3 mg/kg dose of fasudil resulted in a decrease of 3% in the Ao pressure and a 9% decrease in pulmonary artery pressure. The 1.0 mg/kg dose of fasudil resulted in a decrease of 10% in the Ao pressure and 17% in the pulmonary artery pressure. From the above results, we chose the 0.3 mg/kg dose of fasudil for future experiments.

#### 3.3 Effect of CAR Alone

Three separate experiments were performed in which CAR was administered alone at a dose of 1.5 mg/kg. Ao and RV pressures were unchanged during 30 minutes of monitoring (results not shown).

#### 3.4 Safety

During each experimental procedure, the animals had no episodes of life-threatening systemic hypotension, hypoxemia, or apparent allergic reactions to CAR or fasudil. Recovery from anesthesia was uncomplicated and uneventful. In between experimental procedures, the animals had no respiratory or cardiovascular symptoms. They continued with normal feeding, stooling and urine output, and physical activity.

#### 3.5 Fasudil Alone vs. Fasudil with CAR

**Fig. (2):**
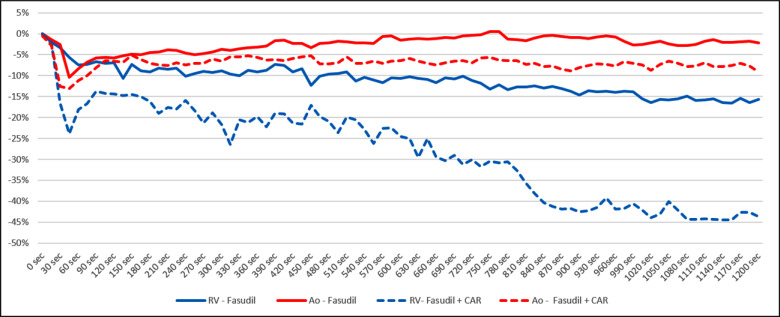
Representative percent decrease of RV and Ao systolic pressures for fasudil alone and fasudil + CAR. **Legend:** Ao (red) and RV (blue) systolic pressures following the administration of fasudil (0.3 mg/kg) alone (solid lines) and fasudil after CAR (1.5 mg/kg) (broken lines). Time is on the X-axis, in seconds. The percent decrease in systolic pressure is on the Y-axis. Ao = Aortic, RV= Right Ventricular.

**Fig. (3):**
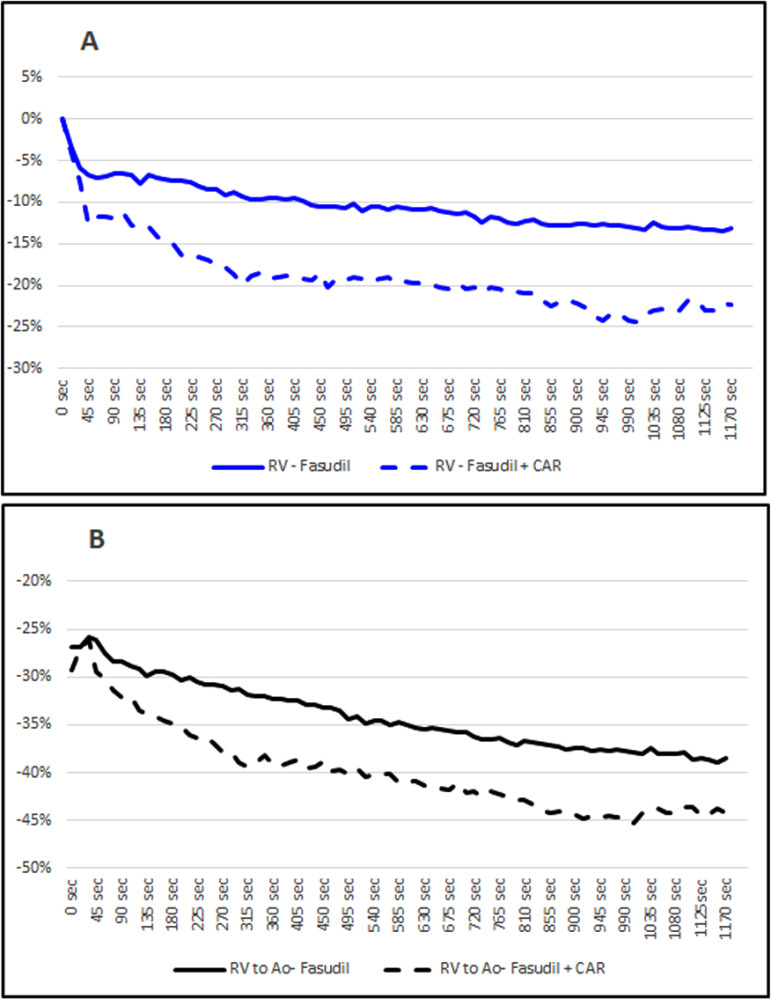
Change in RV and Ao pressure for all experiments. **Legend:** Average percent change in RV systolic pressure (**A**, blue curves) and average percent difference in RV/Ao systolic pressure ratio (**B**, black curves) comparing fasudil (0.3 mg/kg) alone (solid lines) with fasudil + CAR (1.5 mg/kg) (broken lines) for all the experiments. Time is on the X-axis in seconds, and percent change in RV/Ao pressure is on the Y-axis. Ao=Aortic, RV=Right Ventricular.

We performed a total of 16 experiments on 3 animals, 8 with fasudil alone and 8 with fasudil combined with CAR. Fasudil alone caused a 12% mean decrease, while fasudil + CAR caused a 22% decrease in the systolic RV pressure (p < 0.0001). Fasudil alone caused an average decrease of 34%, while fasudil + CAR caused an average decrease of 40% in the RV systolic pressure in relation to the Ao systolic pressure (RV/Ao pressure ratio) (p < 0.0001) (Figs. **2** and **3**). The difference in mean area under the curve for the percent change of RV pressure from baseline for fasudil alone vs. fasudil + CAR was significant (-1.84 *vs*. -3.89, p < 0.0001). The difference in mean area under the curve for the RV/Ao pressure ratio for fasudil alone *vs*. fasudil + CAR was also significant (5.17 *vs*. 6.00, p < 0.0001).

## 4 DISCUSSION

Despite improvements in morbidity and mortality over the past three decades, pulmonary hypertension outcome continues to be poor, with a mean survival of six years [2, 5, 6]. Fourteen medications have been approved to treat pulmonary hypertension [7]. Systemic hypotension and other side effects have limited the use of pulmonary hypertension medications. A more selective method of targeting pulmonary hypertension medications to the pulmonary vasculature would be beneficial. CAR is a cyclic peptide that was shown to preferentially bind to pulmonary vascular tissue in rats with monocrotaline-induced pulmonary hypertension [8]. CAR was subsequently shown to improve the selectivity for vascular vasodilatory effects on pulmonary circulation compared with the systemic circulation of fasudil, sildenafil, and imatinib in rats [3].

Studies have demonstrated that fasudil, a Rho kinase inhibitor, has significant pulmonary vascular effects in mouse and rat pulmonary hypertension models. Fasudil caused a decrease in vasoconstriction and vascular remodeling in response to hypoxia in mice and monocrotaline in rats [9, 10]. Furthermore, fasudil attenuated the rise in pulmonary arterial pressure and reversed pulmonary vasoconstriction caused by nitric oxide synthase antagonists in chronically hypoxic mouse lungs, and it also lowered acute hypoxia-mediated contraction of isolated rat pulmonary artery segments [9, 11]. Oka *et al.* [12] showed that in rats, the Rho kinase inhibitor fasudil acutely and effectively reduced the end-stage angioproliferative pulmonary vascular pathology, resembling the pathologic changes observed in severe human pulmonary arterial hypertension.

Despite strong preclinical data, there are limited reports describing the effects of fasudil in patients with pulmonary hypertension. Fukumoto *et al.* [13] and Ishikura *et al.* [14] reported that intravenous fasudil (30 mg for 30 minutes) decreased pulmonary artery pressure, increased cardiac index, and reduced pulmonary vascular resistance in 9 and 8 patients, respectively. Jiang *et al.* [15] reported the acute effects of intravenous fasudil (30 mg over 30 min) in 50 patients; mean pulmonary artery pressure decreased from 61.7 to 57.1 mmHg, pulmonary vascular resistance decreased from 15.9 to 12.9 Wood units, and cardiac output increased from 3.6 to 4.1 L/min.

Fujita *et al.* [16] described the effects of inhaled nitric oxide (40 ppm, 10 min) and fasudil (30 mg, 10 min) in 15 patients with pulmonary hypertension. Both nitric oxide and fasudil reduced mean pulmonary arterial pressure and tended to decrease pulmonary vascular resistance but did not affect the cardiac index. Kojonazarov *et al.* [17] reported in 19 patients with high-altitude pulmonary hypertension that intravenous fasudil markedly decreased pulmonary vascular resistance. Li *et al.* [18] and Xiao *et al.* [19] reported acute vasodilator effects of intravenous fasudil (30 mg over 30 min) in 12 and 35 patients, respectively, with congenital heart disease and pulmonary hypertension. Mean pulmonary artery pressure decreased from 65.4 to 57.3 mmHg, and pulmonary vascular resistance decreased from 10.7 to 8.4 Wood units [19]. The left-to-right shunt ratio increased by 16.2%, the systemic vascular resistance decreased by 10.2%, and the pulmonary-to-systemic vascular resistance ratio decreased by 23.9% [18]. Therefore, fasudil has significant acute vasodilatory effects in human studies with relatively small numbers of patients. However, fasudil has not been approved for the treatment of pulmonary hypertension in the United States.

Our study is the first to demonstrate that CAR enhanced the vasodilatory effects of fasudil in pulmonary circulation compared with systemic circulation in a large animal model of acute and chronic pulmonary hypertension. Whereas the same effect of CAR was demonstrated in rats [3]. This study aimed to extend the observations to larger animals and a model with close similarities to human pulmonary hypertension. The European Medicines Agency, the US Food and Drug Administration, and the International Society for Stem Cell Research recommend the use of large animal models to evaluate the efficacy, durability, dose-response, degradation, and safety of advanced therapeutic medicinal products [20, 21]. Moreover, but not without controversy, some have suggested that the predictability of human effects of stem cells and other therapeutic agents may be enhanced if demonstrated in more than one animal model [22]. Furthermore, our microsphere model has significant mechanical, hemodynamic, and pathologic similarities to thromboembolic pulmonary hypertension in humans [23, 24].

There were no adverse acute or chronic effects of CAR or fasudil on animals. They recovered well from anesthesia and had no significant respiratory, cardiac, or other symptoms immediately after or between the vasodilator testing procedures. CAR alone caused no significant effects on Ao or RV pressure. Fasudil caused a rapid decrease in Ao pressure, followed by a progressive recovery to baseline. Fasudil alone resulted in a decrease of 12% in RV systolic pressure, whereas co-administration of CAR with fasudil resulted in a decrease of 22% in RV pressure. Similar results were observed when comparing the change in RV/Ao pressure ratio after fasudil vs. fasudil + CAR.

The co-administration of CAR with pulmonary hypertension medications in humans could result in lower required doses of medications, improved effectiveness, and less systemic hypotension and other adverse side effects. In our study, we used intravenous fasudil. However, other investigators have reported that CAR enhanced the effects of oral and inhaled fasudil on pulmonary hypertension in rats [3, 25-27]. Moreover, sublingual CAR was shown to be as effective as intravenous CAR in enhancing the beneficial pulmonary selective effects of pulmonary hypertensive medications in rats [3].

There are several limitations to our study. We report on only one dose of fasudil and in a limited number of animals. However, it was adequate to demonstrate, with a high degree of statistical significance, that CAR enhanced the effect of this dose of fasudil. Another limitation could be related to our model. We were only able to generate a baseline pulmonary hypertension level of approximately 30-35% systemic level with the repeated infusion of microspheres. Nonetheless, acute administration of the microspheres on the day of vasodilator testing allowed us to attain a pressure of about 2/3-3/4 systemic prior to administering fasudil alone or fasudil + CAR. The fact that CAR was effective at this modest level of chronic pulmonary hypertension, given its previous reports of the effectiveness in other preclinical models with more moderate or severe pulmonary hypertension levels, suggests that CAR may be effective in other experimental large animal models and humans with varying degrees of pulmonary hypertension. Additionally, the fact that CAR and fasudil were effective in lowering acute pulmonary hypertension could suggest a potential but a speculative therapeutic role for the combination of CAR and a pulmonary vasodilator in human acute pulmonary hypertensive crisis.

## CONCLUSION

In conclusion, the co-administration of CAR resulted in a safe, enhanced vasodilatory profile of fasudil in the pulmonary vasculature of a large experimental model of acute and chronic pulmonary hypertension created by infusion of microspheres. We are currently studying the targeted vasodilatory effects of CAR with other pulmonary hypertension medications in the same experimental model. This study adds to the growing body of data suggesting that CAR appears safe and effective in preclinical models and may be ready for trials in patients with pulmonary hypertension.

## AUTHOR’S CONTRIBUTIONS

A. Rothman contributed to the study conception and design, material preparation, data collection, analysis, writing the first draft, and approval of the final manuscript.

H. Restrepo contributed to material preparation, data collection, analysis, revision of drafts, and approval of the final manuscript.

W.N. Evans contributed to data collection, analysis, revision of drafts, and approval of the final manuscript.

V. Sarukhanov contributed to material preparation, analysis, revision of drafts, and approval of the final manuscript.

D. Mann contributed to the analysis, revision of drafts, and approval of the final manuscript.

## Data Availability

The datasets used and/or analyzed during the current study are available from the corresponding author [A.R] upon reasonable request.

## LIST OF ABBREVIATIONS

Ao: = Aortic
RV: = Right Ventricular
RV: = Right Ventricle
